# The divergent impacts of digital engagement on depression: a longitudinal study of competitive resource mechanisms

**DOI:** 10.3389/fpubh.2026.1887437

**Published:** 2026-08-25

**Authors:** Jidong Hu, Lei Liu, Yang Zou

**Affiliations:** 1 School of Marxism, Henan Normal University, Xinxiang, Henan, China; 2 Graduate School of Education, Dalian University of Technology, Dalian, Liaoning, China

**Keywords:** depression, digital engagement, emotional compensation, physiological cost, resource conservation

## Abstract

Digital engagement has become an important factor influencing mental health, yet the complex effects of different usage patterns still require deeper clarification. This study distinguishes digital engagement into instrumental-oriented digital engagement (IE) and affect-oriented digital engagement (AE), and utilizes panel data on the working-age population from the China Family Panel Studies (CFPS) from 2016 to 2022 to examine their associations with depression and their underlying competitive resource mechanisms. The results indicate that IE is associated with higher depression levels, whereas AE is associated with lower depression levels. Mechanism analysis reveals that both types of digital engagement contribute to enhancing psychological mastery resources, but also consume physiological recovery resources such as sleep and exercise. Among them, the physiological loss associated with IE is more likely to offset its psychological gains, whereas AE is more likely to perform an emotional recovery function under conventional intensity levels. Further analysis shows that the association between IE and depression is stronger among populations with high social role stress, and the association between AE and depression exhibits nonlinear characteristics. The study suggests that different digital engagement patterns exhibit asymmetric and competitive resource characteristics. Public health interventions should not simply emphasize reducing digital use, but rather guide individuals to maintain a dynamic balance between instrumental digital loads and moderate emotional recovery.

## 1 Introduction

Digital technology has profoundly reshaped individuals’ living environments and lifestyles, becoming a crucial factor influencing public psychological well-being. Therefore, in-depth research into the association between digital engagement and depression is of significant importance for exploring mental health management in the digital age.

To address this issue, existing research has extensively examined specific dimensions, such as social media use and screen time, yet mixed findings remain. Some studies emphasize that digital involvement may entail psychological risks. Excessive screen time and digital behaviors such as social media use can crowd out users’ real-life activities and time resources (1, 2). This zero-sum dynamic can increase individual psychological burden (3–5). Conversely, other researchers adopt an adaptive value perspective, suggesting that moderate digital use can expand social support and offer new spaces for emotional expression and psychological regulation, thereby improving the digital well-being experience (6, 7). These divergent findings indicate that the psychological consequences of digital engagement can hardly be explained in a unified manner solely by usage levels.

To understand these discrepancies, relevant studies have gradually focused on specific activity contents and modes of engagement. Specifically, existing research has begun to dismantle the assumption that digital engagement is a homogeneous whole, categorizing it based on content into heterogeneous types such as learning, socializing, and entertainment, or distinguishing between active and passive engagement according to user attitudes (8, 9). Relying on these classification frameworks, scholars have further explored the underlying mechanisms of digital engagement, confirming the differentiated effects of diverse pathways such as social capital, self-efficacy, psychological detachment, and time displacement (10). These explorations suggest that the objective form of digital activity is an important factor in shaping its psychological consequences.

In investigating the mechanisms linking digital engagement to depression, the depletion of physiological resources is widely regarded as one of the core pathways. Among these, sleep deprivation is particularly critical (11). Existing research indicates that continuous digital engagement not only compresses essential sleep duration through time displacement, but also induces cognitive and emotional arousal through information interaction, profoundly affecting individual sleep rhythms and sleep quality (12, 13). In addition, the lack of offline physical exercise constitutes another key pathway of physiological depletion (14). This reduction in physical activity, caused by digital time replacement, weakens the protective effectiveness of regular exercise in buffering stress and maintaining physical reserves (15), serving as an important contributor to heightened psychological burden. Conversely, other studies point out that digital engagement serves as an important avenue for individuals to acquire psychological and social resources. Specific digital activities can foster crucial psychological detachment, facilitating immediate emotional recovery (16). Individuals can also leverage digital networks to transcend physical boundaries, thereby maintaining weak-tie social capital and gaining an online sense of belonging (17, 18).

As research has progressed, scholars have successively noted that the functional structure and psychological consequences of digital engagement exhibit life-course characteristics. Because adolescents are in a developmental stage psychologically, their digital behavior focuses heavily on entertainment exposure and social media. Moreover, the underlying mechanisms for this age group tend to center on contexts such as peer recognition, identity development, and parental supervision (19, 20). In contrast, digital engagement among working-age populations is often deeply embedded in multiple social roles. Frequent digital connectivity can accelerate the pace of work while blurring the spatiotemporal boundaries between work and non-work life (21). This implies that adult digital engagement extends beyond screen exposure, intertwining multiple functions such as occupational execution, role maintenance, and emotional compensation. Consequently, the behavioral logic of digital engagement in working-age populations differs substantially from that of adolescents, requiring an explanation of complex psychological mechanisms in light of underlying functional attributes.

Although existing research has deepened the understanding of digital behavior, room for further advancement remains. Existing classifications primarily focus on objective activity content or forms of engagement, with less simultaneous attention paid to the functional orientations and value weights assigned to them by individuals. Particularly for working-age populations, objectively similar digital activities may serve distinct functions, and mere behavioral frequency can hardly represent the true depth of engagement. In this context, value weights reflect individuals’ priority sequences in allocating resources. Therefore, combining objective behavioral frequency with subjective importance promises to more effectively identify digital engagement and its psychological outcomes, a logic that coincides with social action theory.

In light of this, the present study categorizes digital engagement into instrumental-oriented digital engagement (IE) and affect-oriented digital engagement (AE) based on functional logic, and incorporates value weights into empirical measurement with reference to social action theory. Furthermore, grounded in conservation of resources (COR) theory, a competitive analytical framework of “resource gain—resource loss” is constructed to explore the explanatory pathways connecting digital engagement to depression.

To examine the longitudinal associations between IE, AE, and depressive symptoms among the working-age population, this study utilizes four waves of data from the China Family Panel Studies (CFPS) from 2016 to 2022 and proposes the following hypotheses: (1) IE is positively associated with depression, whereas AE shows an opposite association; (2) in terms of resource structure, the pathway of physiological recovery resource loss is relatively dominant for IE, whereas the effect on psychological mastery resources is more prominent for AE. Through this design, this study aims to enrich empirical observations of digital behavior and provide certain support for understanding resource allocation in complex digital interactions.

## 2 Theoretical analysis and hypotheses

Categorizing digital use based on instrumental information acquisition and socio-emotional regulation is conducive to analyzing the characteristics of digital use under different functional attributes (22). However, an individual’s digital engagement is not merely a record of objective behavior; rather, it is an organic integration of external behaviors and the internal value assessments they carry (23). Based on this logic, individual digital engagement can be further categorized into IE and AE. Specifically, IE integrates the characteristics of purposive-rational and value-rational actions. It exhibits a relatively strong goal orientation and primarily serves task completion and self-improvement (24). AE, in contrast, corresponds to affective action, focusing on immediate emotional experiences, entertainment and relaxation, and psychological detachment (25).

According to COR theory, digital engagement represents a process of resource investment in which individuals mobilize limited time and energy to achieve expected goals. This investment subsequently triggers a dual effect of resource gain and resource loss: on the one hand, task achievement or emotional regulation during digital engagement can enhance individuals’ sense of control over their current situation, thereby accumulating psychological mastery resources; on the other hand, continuous digital engagement can crowd out sleep and physical exercise, weakening individuals’ physiological recovery resources. The relative strength of resource gain and loss determines an individual’s net resource gain or net resource loss, thereby shaping its association with depressive symptoms (26).

Within this resource structure, psychological mastery resources and physiological recovery resources constitute two core elements with distinct functions. Psychological mastery resources refer to internal resources that enable individuals to maintain action orientation and continuously advance goals in uncertain environments, the core of which is an individual’s belief in control over their current situation. When this sense of control extends from current actions to future goal pursuits, it manifests as positive expectations that individuals believe they can still improve their current situation. Based on this logic, individual confidence in the future constitutes an empirical expression of psychological mastery resources across the temporal dimension. In contrast, physiological recovery resources are resources used by individuals after continuous activity and stress exposure to repair fatigue and restore energy, as well as to maintain behavioral functioning. They are primarily reflected in an individual’s physical strength, energy, and state of physiological regulation. Generally speaking, sleep and physical exercise are important avenues for individuals to replenish and maintain physiological recovery resources, and their displacement implies a weakening of the individual’s resource recovery process.

**Figure 1:**
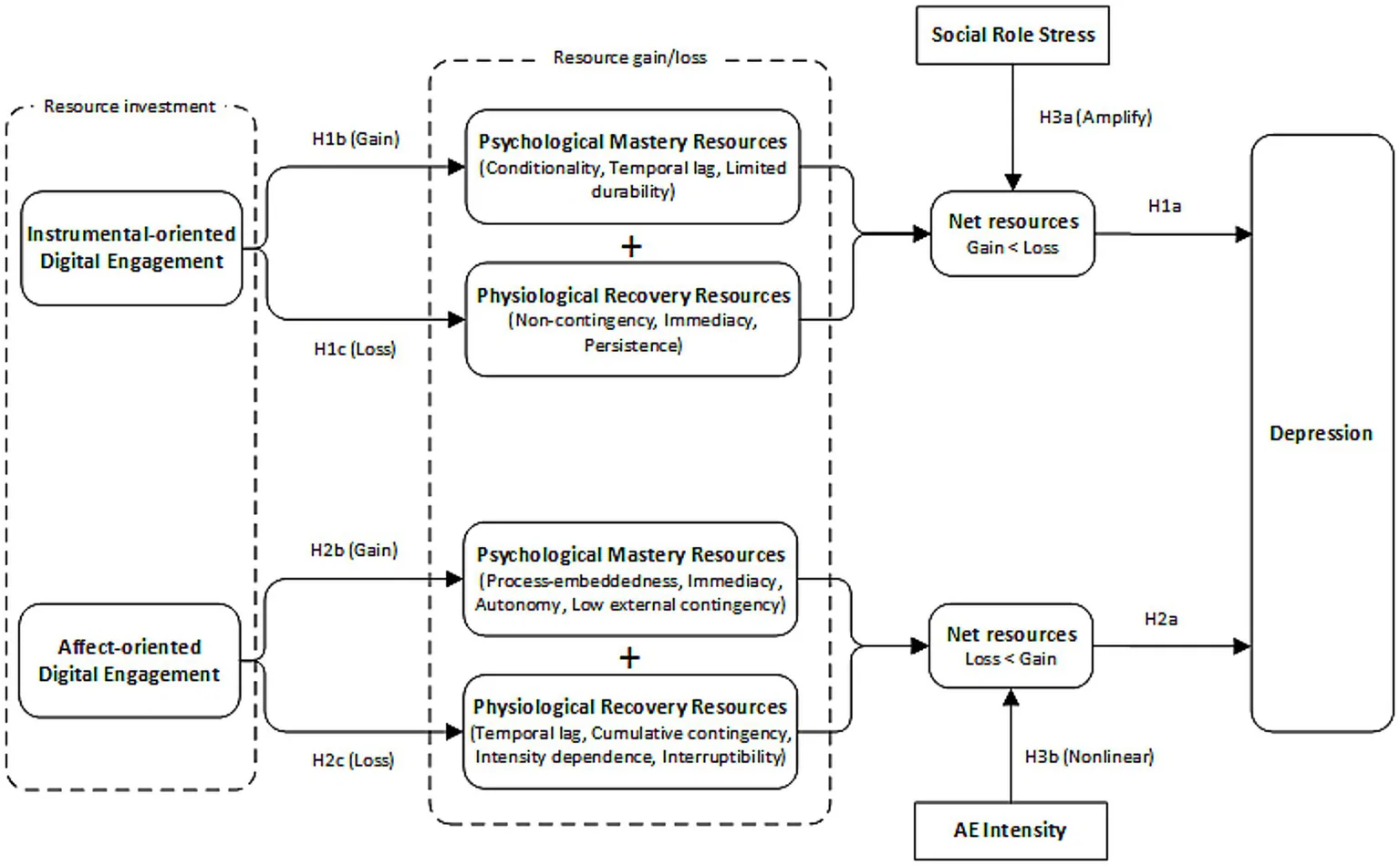
Conceptual diagram.

Although resource investments in both IE and AE trigger resource gain and loss, differences in their underlying behavioral logics reshape the relative advantage of these two pathways. This asymmetry in the weighting of resource gain and expenditure yields distinct net resource outcomes and associations with depression (Figure 1). Grounded in this framework, the following sections will analyze the competitive resource pathways of IE and AE, respectively.

### 2.1 The competitive resource pathway of IE

In a digital society, IE can be understood as a resource investment behavior engaged in by individuals to complete tasks and maintain socioeconomic functioning, often exhibiting a strong goal orientation. When individuals assign higher importance to such activities, IE is more likely to be prioritized in daily actions and lose the elasticity to pause or disengage at any time. To maintain task progress, individuals need to continuously invest time and energy to cope with high-intensity task loads. This continuous and mandatory resource investment triggers a dual resource pathway.

On the one hand, IE contributes to the accumulation of psychological mastery resources in individuals. IE typically encompasses actions such as information retrieval, knowledge learning, and work processing. In this process, individuals enhance their capacity to understand and predict the external environment, while also gaining efficacy experiences from positive feedback between actions and results. This reinforces the belief in control that individuals can intervene in their current circumstances, extending to subsequent goal pursuits (27). As psychological mastery resources accumulate, individuals can take coping measures more proactively when facing uncertainties to maintain goal continuity, thereby alleviating feelings of helplessness regarding their current situation (28).

On the other hand, continuous investment in IE is also accompanied by the loss of physiological recovery resources. IE often involves high cognitive loads and goal management pressures. Because individuals endorse the importance of such activities, to maintain task progress and immediate responsiveness, they are more likely to breach fixed temporal boundaries, allowing activities to extend into periods originally reserved for rest. Constrained by performance deadlines, sleep and physical exercise, lacking immediately visible returns, are more easily compressed in time allocation. High-intensity IE not only crowds out baseline sleep duration but also compresses the time and energy available for regular exercise, thereby weakening opportunities for individuals to repair fatigue and regulate stress (29). Therefore, the absence of recovery activities implies that individuals face continuous losses of physiological recovery resources, which are subsequently associated with higher levels of depression.

However, constrained by the asymmetry in the resource transformation logic of IE, it generally exhibits an asymmetric pattern dominated by resource loss. The acquisition of psychological mastery resources relies on substantive task progress and action feedback, requiring a prolonged investment process and entailing the risk of failure. In addition, the characteristics of IE constrain the space for proactively interrupting resource investment, and the benefits it yields are often contingent upon specific contexts (30). Consequently, the acquisition of psychological mastery resources is characterized by lag, conditionality, and difficulty in stable continuation. Conversely, the loss of physiological recovery resources is immediate and continuous. When individuals become involved in IE, the spatiotemporal compression of sleep and exercise may subsequently occur, and this does not depend on goal realization as a precondition. The physiological overdraft caused by a lack of rest and exercise, however, diffuses into overall behavioral and emotional regulation, continuously accumulating under the constraint of lacking interruption elasticity. These differences in realization probability, timing of occurrence, and scope of duration tilt the resource balance of IE toward the loss side, giving rise to net resource loss and correlating with higher depression (31).

*H1a*: IE is generally positively associated with individual depression levels.

*H1b*: IE is positively associated with individuals’ psychological mastery resources, which in turn are associated with lower depression levels.

*H1c*: IE is negatively associated with individuals’ physiological recovery resources, which in turn are associated with higher depression levels.

### 2.2 The competitive resource pathway of AE

Unlike IE, AE primarily revolves around emotional experiences, entertainment, relaxation, and psychological detachment. The core value of such activities often generates within the process of digital engagement itself, without requiring external goal achievement as a precondition. Therefore, AE is typically free from the coercive drive of real-world performance or external responsibilities, giving individuals a high degree of autonomy when investing resources. Nevertheless, AE still occupies a certain amount of time and energy, thereby triggering both resource gain and loss.

AE constitutes an effective avenue for individuals to replenish psychological mastery resources. Recreational digital activities are usually characterized by autonomous choice and immediate feedback. In this process, individuals can adjust the content and pace of engagement according to their own needs, thereby temporarily interrupting connections with real-world stressors (32). The resulting psychological detachment not only alleviates negative emotions, but also allows individuals, within self-directed digital spaces and times, to further consolidate their sense of regulatory control over emotions and behaviors. The efficacy of this immediate feedback reinforces individuals’ beliefs in control, assuring them of their ability to regulate their current state and subsequently maintaining positive expectations for advancing future goals and improving current situations (33). As a result, through AE, individuals acquire immediate psychological mastery resources, which subsequently reduces depression risk.

AE also entails the issue of physiological recovery resource loss. Immersive experiences of recreational digital content often exhibit strong stickiness, causing individuals’ digital involvement to cross flexible boundaries and thereby encroach upon the objective spatiotemporal domain of basic sleep and regular exercise. This spatiotemporal compression limits opportunities for individuals to repair fatigue and restore energy, making it difficult for physiological recovery resources to be fully replenished. As fatigue accumulates, individuals’ ability to maintain emotional stability and cope with external demands may also decline, which is subsequently associated with higher levels of depression.

The dual resource pathways triggered by AE exhibit noticeable differences in timing and conditions. The acquisition of psychological mastery resources is embedded within the participation process itself. With the help of high-autonomy psychological detachment, individuals can rapidly establish a sense of control over their emotional states, and psychological gains may subsequently take shape. The value generation of this dividend is independent of the verification of external goals. In comparison, the loss of physiological recovery resources manifests cumulative lagging characteristics. Related physiological costs accumulate only after entertainment activities persistently and deeply compress the time and space meant for recovery activities (34). In addition, because AE is less constrained by performance or responsibility, individuals possess a higher elasticity to interrupt their behavior. Upon perceiving physical fatigue or achieving expected emotional compensation, individuals can terminate AE, thereby limiting the unrestrained outflow of physiological costs (35). Therefore, under conventional participation conditions, the resource balance in AE tilts toward the gain side, forming an overall net resource gain and correlating with lower depression.

*H2a*: AE is generally negatively associated with individual depression levels.

*H2b*: AE is positively associated with individuals’ psychological mastery resources, which in turn are associated with lower depression levels.

*H2c*: AE is negatively associated with individuals’ physiological recovery resources, which in turn are associated with higher depression levels.

### 2.3 Boundary conditions of digital engagement

The competitive resource pathways of IE and AE are heterogeneous. The resource transformation effects of digital engagement are deeply embedded in individuals’ real-world role structures. Taking the sandwich generation, who simultaneously bear child-rearing and caregiving responsibilities for older adults, as an example, multiple caregiving responsibilities pre-compress individuals’ disposable resources and recovery space, thereby constituting a structural role load. In this context, IE often degenerates into an extension of multiple responsibilities. The accomplishment of digital tasks is mostly limited to maintaining the basic operation of the existing role structure, making it difficult to foster incremental mastery efficacy. Meanwhile, the limited recovery time and space of this group are more easily subject to further compression, leading to the accumulation of fatigue and role conflict. Consequently, social role stress further imbalances the resource pathway of IE, thereby heightening depression risk.

The immediate feedback characteristic of AE implies that its boundary is closely related to behavioral involvement intensity. Within a moderate range of participation, relying on high autonomy, individuals can efficiently obtain emotional compensation to buffer against depression. However, as involvement intensity rises, this resource balance changes. On the one hand, repetitive AE diminishes the marginal returns of emotional compensation, and psychological detachment may shift from brief recovery to persistent avoidance, causing the accumulation of mastery beliefs to slow down. On the other hand, mechanisms such as algorithmic recommendations and instant rewards tend to prolong participation time and weaken the capacity for proactive interruption, resulting in continuous compression of recovery activities. When continuous involvement is accompanied by diminished control and functional impairment, AE may evolve from autonomous recovery into problematic use. At this point, the resource status of AE dynamically shifts, and its relationship with depression exhibits a nonlinear pattern.

*H3a*: Social role stress strengthens the positive association between IE and individual depression levels.

*H3b*: The association between AE and individual depression levels exhibits a U-shaped nonlinear pattern.

## 3 Materials and methods

### 3.1 Study design, data source, and participants

Based on four waves of data from the CFPS in 2016, 2018, 2020, and 2022, this study conducted a secondary longitudinal panel analysis, with an observation window spanning from 2016 to 2022. The CFPS is a biennial longitudinal survey implemented by Peking University, covering 25 provinces, municipalities, and autonomous regions. Its sampling was conducted sequentially down to the county, community, and household levels, encompassing comprehensive indicators such as economic activities, physical and mental health, and social behaviors. The longitudinal tracking characteristics of this database are suitable for examining changes in digital engagement and depressive symptoms over time. Regarding the choice of years, China’s mobile internet and digital algorithms began to surge starting in 2016. Objectively, this contributed to the differentiation of individual digital engagement, providing a factual and empirical foundation for the present study.

The analytical sample was restricted to the working-age population aged 18 to 59 years. The digital behaviors of minors are highly dependent on factors beyond individuals’ control, such as parental supervision and school discipline. Digital engagement among older adults is often inevitably and deeply intertwined with specific factors such as labor market exit, the digital access divide, and social isolation. Furthermore, depressive symptoms in minors and older adults are more susceptible to specific physiological and developmental psychological factors. These population characteristics are misaligned with the theoretical focus of this study, and may also introduce confounding bias and unobserved heterogeneity into the empirical analysis.

In the survey items for each wave, responses such as “do not know, refuse to answer, and not applicable” were coded as missing values. When constructing variables, a complete-case analysis approach was employed to handle missing values, excluding observations with missing data on any required variable. Subsequently, the four waves of data were merged into a long-format unbalanced panel dataset.

In summary, the baseline analysis comprised 34,198 person-year observations involving 12,605 unique individuals. Tables 1, 2 report the sample screening procedure and descriptive statistics for each wave of the sample. The primary analytical estimand of this study is the within-person association between changes in digital engagement intensity and changes in depressive symptoms among the working-age population, after controlling for individual time-varying and time-invariant factors as well as year fluctuations.

**Table 1:** Sample selection criteria.

Step	Sample definition	Observations	Unique individuals
1	Unbalanced panel after merging 4 waves	125,080	49,740
2	Exclude minors and older adults	86,292	35,242
3	Valid depression	80,383	33,127
4	Valid depression, IEI, and AEI	47,322	23,088
5	Valid depression, IEI, AEI, and control variables	42,954	21,321
6	Baseline regression model: exclude missing values and single-period observations	34,198	12,605

**Table 2:** Sample statistics by wave.

Year	Initial observations	Excluding minors and older adults	Observations in the baseline model
2016	36,892	27,200	7,233
2018	32,641	22,053	8,121
2020	28,546	19,089	9,596
2022	27,001	17,950	9,248
Total	125,080	86,292	34,198

The CFPS study was approved by the Biomedical Ethics Committee of Peking University.

### 3.2 Variables design

#### 3.2.1 Dependent variable

This study uses the 8-item Center for Epidemiologic Studies Depression Scale (CES-D-8) from the CFPS to measure individuals’ depression levels. The 8 items across all waves are scored on a scale from 1 to 4. Among them, the two items, “I felt happy” and “I enjoyed life,” serve as indicators of positive emotions; we reverse-score the responses to these two items. Ultimately, the 8 items are summed to obtain the individual’s depression level, with the total score ranging from 8 to 32. A higher value indicates a higher level of self-rated depression. It is worth noting that if an individual has missing values for any of the items, no score is calculated for that individual. Additionally, we calculated the Cronbach’s alpha for the CES-D-8. The alpha coefficients for the respective waves are 0.724, 0.801, 0.766, and 0.754, indicating that the CES-D-8 possesses a generally stable internal consistency reliability.

#### 3.2.2 Independent variables

The core explanatory variables of this study are instrumental-oriented digital engagement intensity (IEI) and affect-oriented digital engagement intensity (AEI). Both are indicators of digital engagement intensity weighted by subjective importance, which approximately characterize the degree of an individual’s behavioral investment in the corresponding domains.

In the classification of digital behaviors, the domains of digital activities provide empirical clues for observing behavioral functions. Work, learning, and life management activities in the CFPS usually revolve around task completion, problem solving, and the maintenance of socioeconomic functions; entertainment-oriented digital activities, such as online gaming and watching short videos, are more likely to serve emotional gratification and stress relief. Therefore, following the empirical approach of Jia et al. (36), this study uses these two types of activities as the primary empirical carriers for IE and AE, respectively.

Considering that digital engagement is an organic integration of observable behaviors and internal value assessments, we use the product of importance and frequency ( $\text{Intensit}y_{j}=\text{Frequenc}y_{j}\times \text{Importanc}e_{j}$ ) as the observable proxy variable for digital engagement intensity. In this context, j represents the set of different types of behaviors. The specific design is as follows.

1. In the subjective intention dimension, we use the item “the importance of the internet to different domains j” for measurement, where larger values indicate higher importance. In the behavioral frequency dimension, the measurement scale for “the frequency of using the internet for different behaviors j” changed starting in 2020. To maintain consistency across the panel data, we assigned a value of 0 to the responses “2–3 times a month,” “once a month,” “once every few months,” and “never” in the first two waves of data; a value of 1 to “3–4 times a week” and “1–2 times a week”; and a value of 2 to “almost every day.” In the latter two waves of data, we assigned a value of 0 to the response “no” for “whether j was performed in the past week,” a value of 1 to the response “no” for “whether j was performed every day in the past week,” and a value of 2 to “yes.”
2. Based on the “value-behavior” model, we obtained the digital engagement intensity for different domains j, respectively.

Based on the digital engagement intensity within a specific domain, this study references the empirical approach of Sparks et al. (37) and uses the maximum value aggregation method to obtain the proxy variables for IEI and AEI, respectively.


$$\text{Intensit}y_{j}=\max(\text{Intensit}y_{j1},\text{Intensit}y_{j2},\cdots ,\text{Intensit}y_{\mathrm{jn}})$$ (1)

This approach primarily reflects the intensity of the dominant domain in an individual’s digital activities. This aligns relatively well with this study’s theoretical definition of digital engagement. As actors with limited time and psychological resources, individuals usually do not maintain a balanced investment across all domains of the same type of digital activity; rather, they are more likely to concentrate their resources in domains most relevant to their social roles or practical needs. For example, the IE of students and workers may primarily manifest as online learning and online work, respectively. Similarly, AE may also be concentrated in a dominant channel, such as online gaming or short video use. Based on this, maximum value aggregation is suitable for capturing the intensity of the dominant domain of a specific type of digital engagement.

Furthermore, IEI and AEI are more akin to formative or composite indicators (38, 39). In empirical data, role differences and temporal mutual exclusivity may exist among different domains of digital activities, resulting in low or zero values in certain domains. In this context, if the mean aggregation method is used, the low engagement intensity of non-dominant domains may dilute the engagement intensity of the dominant domain; conversely, summation aggregation may misjudge cross-domain engagement breadth as engagement intensity, causing moderate engagement across multiple domains to be misidentified as high-intensity engagement in a single dominant domain. In comparison, maximum value aggregation is more consistent with the engagement intensity of the dominant domain.

#### 3.2.3 Construct validity of explanatory variables

In real-world digital contexts, a single digital behavior is often intertwined with multiple attributes, and its functional boundaries are not absolutely clear. However, constrained by individuals’ core demands and resource allocation, the same behavior typically possesses a relatively dominant value assessment in specific contexts (40). Based on this logic, the classification of IE and AE in this study is an empirical approximation based on the primary functional attributes of digital activities.

Following the logic of social action, digital engagement should be understood as an organic integration of objective behavior and value assessment. Therefore, we construct proxy variables along the two dimensions.

Usage frequency is the proxy variable for external behavior, which reflects the individual’s actual degree of participation in specific digital activity domains. A higher usage frequency implies that individuals have invested more behavioral resources in such digital activities.

In the dimension of value assessment, we use importance as the proxy indicator. Drawing on the logic of Subjective Task Value in Expectancy-Value Theory, an individual’s selection of and investment in specific activities usually depend on their subjective value assessment of those activities (41). The degree of importance that individuals assign to a certain type of digital activity can be understood as an empirical mapping of their priority value judgments in that domain (42). Therefore, importance evaluation can serve as an empirical proxy variable for value assessment in digital behaviors.

Based on usage frequency and importance, we adopt a multiplicative model to construct an indicator of digital engagement intensity that integrates both objective behavior and value assessment. Authentic digital engagement is a unity of external behavioral investment and internal value identification, both of which possess irreplaceable complementarity. The multiplicative logic aligns relatively well with this premise. Only when both external behavior and subjective importance reach relatively high levels simultaneously can the behavior be regarded as having a high action intensity. Consequently, under the limitations of the CFPS data, this study constructs the proxy variables for IEI and AEI by combining the domains of digital activities, usage frequency, and subjective importance.

It is worth noting that the maximum value aggregation method may introduce risks, such as amplifying the impact of extreme values and reducing indicator stability. Therefore, the subsequent sections will employ alternative classification standards and replace the aggregation method to conduct sensitivity analyses.

#### 3.2.4 Mediating variables

Individual psychological mastery resources and physiological recovery resources serve as the mediating variables in this study. Combining with survey items, we selected the item “confidence in one’s future” (ranging from 1 to 5 to indicate increasing levels of confidence) as a proxy indicator. To some extent, this indicator reflects individuals’ positive expectations regarding future circumstances and goal realization.

It should be noted that confidence in the future may be conceptually close to the “feeling hopeless about life” item in depression. To this end, we performed supplementary analyses. The correlation coefficient between confidence in the future and depression was −0.269; the VIF in the model including confidence in the future was 1.01; and the variance explained by the first principal component in Harman’s single-factor test was 22.36%. These results do not indicate severe empirical overlap between the two or a scenario dominated by a single common factor, providing preliminary support for using confidence in the future as a proxy variable.

Regarding physiological recovery resources, we simultaneously use sleep duration and exercise duration as proxy indicators. Specifically, we use two questions: “how many hours do you usually sleep per day” and “total duration of physical exercise in the past week.” Because the questioning method for exercise duration was adjusted in different years, this study uniformly converts it into weekly exercise duration. In 2016 and 2018, the question asked for the respondents’ total exercise duration in the past week, which can be used directly. In 2020 and 2022, the questions asked for the exercise frequency and duration per session over the past 12 months. We first convert the exercise frequency categories into the number of weekly exercise sessions, then multiply this by the minutes per exercise session and divide by 60, thereby obtaining the approximate weekly exercise duration. When converting to weekly exercise frequency, an average of less than 1 time per month is assigned a value of 0.125; an average of 1 or more times per month but less than 1 time per week is assigned 0.5; averages of 1 to 2 times and 3 to 4 times per week are assigned their respective mean values. To reduce the impact of extreme values, this study winsorizes the weekly exercise duration at the 1st and 99th percentiles by year, and further standardizes it within each year.

#### 3.2.5 Control variables

We control for marital status, employment status, education level, smoking, and alcohol consumption. The construction situation of each variable can be found in Appendix Table A.

### 3.3 Statistical analysis

#### 3.3.1 Primary fixed-effects model

This study constructs a Two-Way Fixed Effects (TWFE) model, specified as follows:


$$\text{Depressio}n_{\mathrm{it}}=\beta_{1}\mathrm{IE}I_{\mathrm{it}}+\beta_{2}\mathrm{AE}I_{\mathrm{it}}+\text{γControl}s_{\mathrm{it}}+\mu_{i}+\lambda_{t}+\varepsilon_{\mathrm{it}}$$ (2)

In Equation 2, $\mu_{i},\lambda_{t}$ represent individual and time fixed effects, respectively. $\varepsilon_{\mathrm{it}}$ is the random error term. Standard errors were clustered at the community level to address potential correlation among observations within the same community. $\text{Control}s_{\mathrm{it}}$ denote time varying control variables.

Because this study focuses on the within-individual longitudinal association between digital engagement and depression, we employed an individual and year two-way fixed effects model to identify information primarily stemming from individual changes over time. Under this specification, applying survey weights would alter the estimation target and shift the results closer to a weighted population average association. As this does not align with the analytical level of interest in this study, survey weights were not applied in the regression analyses, and observations with only a single valid wave were excluded.

#### 3.3.2 Sensitivity and robustness analyses

To test the robustness of the primary findings, we performed several sensitivity and robustness analyses.

1. To examine the impact of the construction method of digital engagement intensity on the estimation results, we replaced the maximum aggregation method in the benchmark model with mean aggregation and sum aggregation methods, subsequently re-estimating the models.
2. Considering potential boundary ambiguity issues in the classification of digital activities, we conducted sensitivity analyses using alternative categorization methods.
3. We further conducted robustness tests by adjusting the time span and employing two-way clustered standard errors.

Lagged dependent-variable model. We incorporated prior-wave depressive symptoms into the model to examine whether the association between digital engagement and current depressive symptoms remains stable after controlling for baseline depression levels. Specifically, as shown in Equation 3:


$$\begin{array}{c} \text{Depressio}n_{i,t}=\text{θDepressio}n_{i,t-1}+\beta_{1}\mathrm{IE}I_{i,t-1}+\beta_{2}\mathrm{AE}I_{i,t-1}\\ +\text{γControl}s_{i,t-1}+\lambda_{t}+\varepsilon_{i,t} \end{array}$$ (3)

1. Placebo tests were conducted by randomly permuting IEI and AEI, respectively, to assess whether the benchmark coefficients differ markedly from the coefficient distribution under random exposure assignment.
2. We used the community-level mean IEI and AEI excluding the individual themselves as instrumental variables, incorporating instrumental variable analysis into our robustness checks.

#### 3.3.3 Exploratory pathway and boundary analyses

We adopted a semi-longitudinal mediation model to conduct exploratory pathway analysis. TAs shown in Equations 4 and 5:


$$M_{i,t}=a_{1}\mathrm{IE}I_{i,t-1}+a_{2}\mathrm{AE}I_{i,t-1}+\text{γControl}s_{i,t-1}+\mu_{i}+\lambda_{t}+\varepsilon_{i,t}$$ (4)

$$\begin{array}{c} \;\text{Depressio}n_{i,t}=c_{1}^{\prime }\mathrm{IE}I_{i,t-1}+c_{2}^{\prime }\mathrm{AE}I_{i,t-1}+\sum_{k=1}^{3}b_{k}M_{k,i,t}\\ +\text{γControl}s_{i,t-1}+\mu_{i}+\lambda_{t}+\varepsilon_{i,t} \end{array}$$ (5)

This pathway analysis is established on the sequential ignorability assumption. The 95% confidence intervals (CI) for indirect effects were estimated using Monte Carlo simulation methods. Because the resource variables and depressive symptoms were observed within the same survey wave, results from this part provide semi-longitudinal proxy evidence for the competitive resource mechanisms.

To analyze the boundary role of social role stress, we constructed interaction-term models using the sandwich generation group as a proxy categorical indicator, specified as follows:


$$\begin{array}{c} \text{Depressio}n_{\mathrm{it}}=\beta_{1}\mathrm{IE}I_{\mathrm{it}}^{\prime }+\beta_{2}\mathrm{AE}I_{\mathrm{it}}^{\prime }+\beta_{3}\mathrm{St}r_{\mathrm{it}}+\beta_{4}(\mathrm{IE}I_{\mathrm{it}}^{\prime }\times \mathrm{St}r_{\mathrm{it}})\\ +\beta_{5}(\mathrm{AE}I_{\mathrm{it}}^{\prime }\times \mathrm{St}r_{\mathrm{it}})+\sigma_{\mathrm{it}} \end{array}$$ (6)

$$\sigma_{\mathrm{it}}=\text{γControl}s_{\mathrm{it}}+\mu_{i}+\lambda_{t}+\varepsilon_{\mathrm{it}}$$ (7)

In Equations 6 and 7, $\mathrm{IE}I_{\mathrm{it}}^{\prime }$ and $\mathrm{AE}I_{\mathrm{it}}^{\prime }$ represent the results after sample mean centering, and $\mathrm{St}r_{\mathrm{it}}$ is the dummy variable for social role stress. In addition, we constructed a squared term $\mathrm{AE}I_{\mathrm{it}}^{\prime 2}$ using $\mathrm{AE}I_{\mathrm{it}}^{\prime }$ , and incorporated $\mathrm{IE}I_{\mathrm{it}}^{\prime }$ , $\mathrm{AE}I_{\mathrm{it}}^{\prime }$ , and $\mathrm{AE}I_{\mathrm{it}}^{\prime 2}$ into the model to analyze the nonlinear effects of $\mathrm{AE}I_{\mathrm{it}}^{\prime }$ .

#### 3.3.4 Multiple testing and software

The primary confirmatory tests of this study center on the associations between IEI, AEI, and depressive symptoms. Sensitivity and robustness analyses were primarily utilized to examine the robustness of the main findings, whereas pathway analyses and boundary condition analyses were exploratory in nature. Considering that subsequent analyses revolved around the same core research question, and that different models mostly drew from the same data source and theoretical setup, this study did not adopt multiple testing adjustment methods such as Bonferroni correction. In addition, we comprehensively evaluated the study findings based on information including directional stability of estimates, effect sizes, statistical uncertainty, and consistency of results across different models. The 95% CI for core results from the sensitivity analyses, robustness analyses, and boundary condition analyses are presented in the Appendix.

Among the aforementioned statistical analyses, the Monte Carlo simulations were calculated in R software, while all other analyses were completed using STATA.

## 4 Results

### 4.1 Descriptive statistics of the sample

As shown in Tables 1, 2, after a series of data exclusion procedures, the baseline analytical sample comprised a total of 34,198 person-year observations from 12,605 unique individuals. Within the observation window of this study, respondents were repeatedly observed approximately 2.71 times on average. The distribution across waves was relatively balanced, showing no severe structural attrition.

Table 3 reports the unweighted and weighted descriptive statistical results. Among them, the weighted results utilized standardized individual cross-sectional weights for each wave to provide population sample characteristics. Overall, no substantial changes were found in the distribution characteristics of unweighted and weighted values for each variable. The mean depressive symptoms score was 13.304 in the unweighted sample and 13.266 in the weighted sample. Standard deviations indicated that a certain degree of variation remained in the sample. Regarding digital engagement, the unweighted means of IEI and AEI were 6.430 and 5.600, while the weighted means were 6.550 and 5.586, suggesting that the magnitude of change in the intensity of both types of digital engagement before and after weighting was small. Across different samples, the mean educational attainment level was generally low with a high standard deviation, reflecting a certain degree of variation in educational background among respondents.

**Table 3:** Descriptive statistics.

Variables	Min	Max	Unweighted			Weighted		
			*N*	Mean	SD	*N*	Mean	SD
Depression	8	32	34,198	13.304	3.660	32,144	13.266	3.708
IEI	0	10	34,198	6.430	3.563	32,144	6.550	3.550
AEI	0	10	34,198	5.600	3.272	32,144	5.586	3.279
Marital status	0	1	34,198	0.791	0.406	32,144	0.748	0.434
Employment status	0	1	34,198	0.813	0.389	32,144	0.829	0.376
Educational level	1	8	34,198	3.824	1.346	32,144	3.952	1.343
Smoking	0	1	34,198	0.291	0.454	32,144	0.296	0.457
Alcohol consumption	0	1	34,198	0.130	0.334	32,144	0.150	0.357

Because the TWFE model primarily relies on information from within-individual changes over time, we further examined the within-individual variations in IEI, AEI, and depression. Results in Table 4 show that the within-individual standard deviation for depression was 2.140, while the within-individual standard deviations for IEI and AEI were 2.428 and 2.239, respectively, all indicating a certain degree of within-individual variation. Furthermore, within the observation period, 93.7% of individuals experienced at least one change in depressive symptoms, and 77.9 and 85.7% of individuals experienced at least one change in IEI and AEI, respectively. This indicates that within-individual variation is noticeable, providing an empirical basis for the present study.

**Table 4:** Within-person variation in explanatory variables and dependent variable.

Variables	Overall SD	Between-person SD	Within-person SD	Individuals with any change (%)
Depression	3.661	3.051	2.140	93.7
IEI	3.563	2.679	2.428	77.9
AEI	3.272	2.443	2.239	85.7

### 4.2 Primary fixed-effects results

Column (1) of Table 5 presents the results for IEI and AEI constructed using the maximum aggregation method. Controlling for the other type of digital engagement, AEI was negatively associated with depressive symptoms (−0.009). Conversely, IEI was positively associated with depressive symptoms (0.011), although its 95% CI slightly crossed zero.

**Table 5:** Baseline regression results.

Variables	(1)		(2)	
	Coef. (SE)	95% CI	Coef. (SE)	95% CI
IEI	0.011* (0.006)	[−0.001, 0.023]	0.016* (0.008)	[−0.000, 0.031]
AEI	−0.009** (0.004)	[−0.017, −0.002]	−0.010** (0.004)	[−0.018, −0.002]
Marital status	−0.473*** (0.141)	[−0.749, −0.197]	−0.357*** (0.106)	[−0.566, −0.148]
Employment status	0.045 (0.076)	[−0.104, 0.193]	0.012 (0.021)	[−0.029, 0.052]
Educational level	−0.084* (0.044)	[−0.171, 0.002]	−0.065* (0.034)	[−0.132, 0.001]
Smoking	0.135 (0.145)	[−0.148, 0.417]	0.084 (0.090)	[−0.092, 0.260]
Alcohol consumption	0.116 (0.091)	[−0.062, 0.297]	0.034 (0.025)	[−0.014, 0.083]
TWFE	Yes		Yes	
*N*	34,198		34,198	
Adj R^2^	0.468		0.468	

Standard errors are in parentheses. * *p* < 0.1, ** *p* < 0.05, *** *p* < 0.01. TWFE, Two-Way Fixed Effects.

In terms of effect magnitude, within the sample of this study, when an individual’s IEI increases from lower (P25 = 4) to higher (P75 = 10), expected depressive symptoms increase by 0.066 points. Conversely, if an individual’s AEI increases from lower (P25 = 3) to higher (P75 = 8), the corresponding predicted change in depressive symptoms is −0.045 points. From a practical standpoint, the magnitude of predicted change at the single-individual level is limited. However, considering the high prevalence of digital engagement among the working-age population, such small yet widespread associations may still hold potential cumulative health significance at the population level.

Column (2) further reports the results from the standardized model. The standardized estimate for IEI was positive, but the lower bound of its confidence interval was close to zero, indicating that the statistical evidence remained at a marginal level. AEI, by contrast, exhibited a relatively stable negative association. Overall, Table 5 provides relatively clear and stable statistical evidence for H2a, as well as suggestive evidence for H1a that is consistent with the expected direction. Therefore, we will further verify the associations of IEI and AEI in subsequent analyses.

### 4.3 Exposure-construction sensitivity analyses

To examine whether the research findings depended on specific variable construction methods, we reconstructed IEI and AEI from the aspects of aggregation methods and activity classifications, respectively.

#### 4.3.1 Alternative aggregation

We further employed the mean aggregation method and sum aggregation method to reconstruct the exposure variables and re-estimated the models (Table 6). Under the mean aggregation method, IEI showed a positive association with depressive symptoms (0.018), which was statistically significant at the 5% level; AEI remained significantly negatively associated with depressive symptoms (−0.012). When applying the sum aggregation method, the coefficient for IEI increased to 0.022, though exhibiting marginal significance, while AEI continued to demonstrate a negative association (−0.015).

**Table 6:** Sensitivity and robustness analyses results.

Variables	Sensitivity tests		Alternative classification	Cluster adjustment	Period adjustment	LDV model
	Mean aggregation	Sum aggregation				
IEI	0.018** (0.008)	0.022* (0.012)	0.022*** (0.005)	0.011* (0.007)	0.021** (0.008)	0.018** (0.007)
AEI	−0.012*** (0.002)	−0.015** (0.006)	−0.028*** (0.002)	−0.009*** (0.001)	−0.027*** (0.004)	−0.011** (0.004)
Depression_t-1_						0.490*** (0.008)
Controls	Yes	Yes	Yes	Yes	Yes	Yes
TWFE	Yes	Yes	Yes	Yes	Yes	Yes
*N*	34,198	34,198	34,198	34,198	15,038	20,571

Standard errors are in parentheses. * *p* < 0.1, ** *p* < 0.05, *** *p* < 0.01. TWFE, Two-Way Fixed Effects.

Across different aggregation methods, the association patterns between IEI, AEI, and depressive symptoms did not change substantially. This implies that the baseline results were not driven solely by the maximum aggregation method.

#### 4.3.2 Alternative classification

In the AE categorization, although online gaming serves an instrumental function for a minority of professional esports players or gaming-related practitioners, it generally aligns more closely with recreational attributes in the general population sample. Life affair management in IE may also involve other motivations. Therefore, we excluded life affair management and watching short videos from IE and AE, respectively, yielding a relatively conservative alternative classification.

In Table 6, under the new classification criteria, IEI was positively associated with depressive symptoms (0.022), while the magnitude of the negative association for AEI increased to −0.028, with both estimates exhibiting clearer statistical evidence. This change indicates that among digital activities with relatively clear intentions, the differentiated outcomes between IEI and AEI persist, with more pronounced statistical effects.

### 4.4 Robustness analyses

#### 4.4.1 Clustering adjustment

We further utilized a two-way clustering approach to adjust standard errors simultaneously at both the individual and community levels (Table 6). The effect sizes and statistical significance of the estimates for IEI and AEI remained largely unchanged, suggesting that adjusting the clustering approach did not alter the main association directions.

#### 4.4.2 Adjusting the time span

Considering that the COVID-19 pandemic substantially increased individual use of digital technologies, we excluded the two waves of data from 2020 and 2022. The re-estimated results (Table 6) show that IEI and AEI still maintained statistically significant positive and negative associations with depression, respectively.

#### 4.4.3 Lagged dependent-variable models

Depressive symptoms exhibit temporal persistence, and individuals’ current psychological state may be influenced by prior depression levels. To address this, we employed a lagged dependent variable model. After controlling for prior-wave depressive symptoms, the positive coefficient for IEI increased and was statistically significant at the 5% level; AEI similarly maintained a statistically significant negative association. Under this specification, the estimated directions for both variables remained unchanged, while statistical precision improved, providing evidence of robustness for the baseline results.

#### 4.4.4 Placebo tests

**Figure 2:**
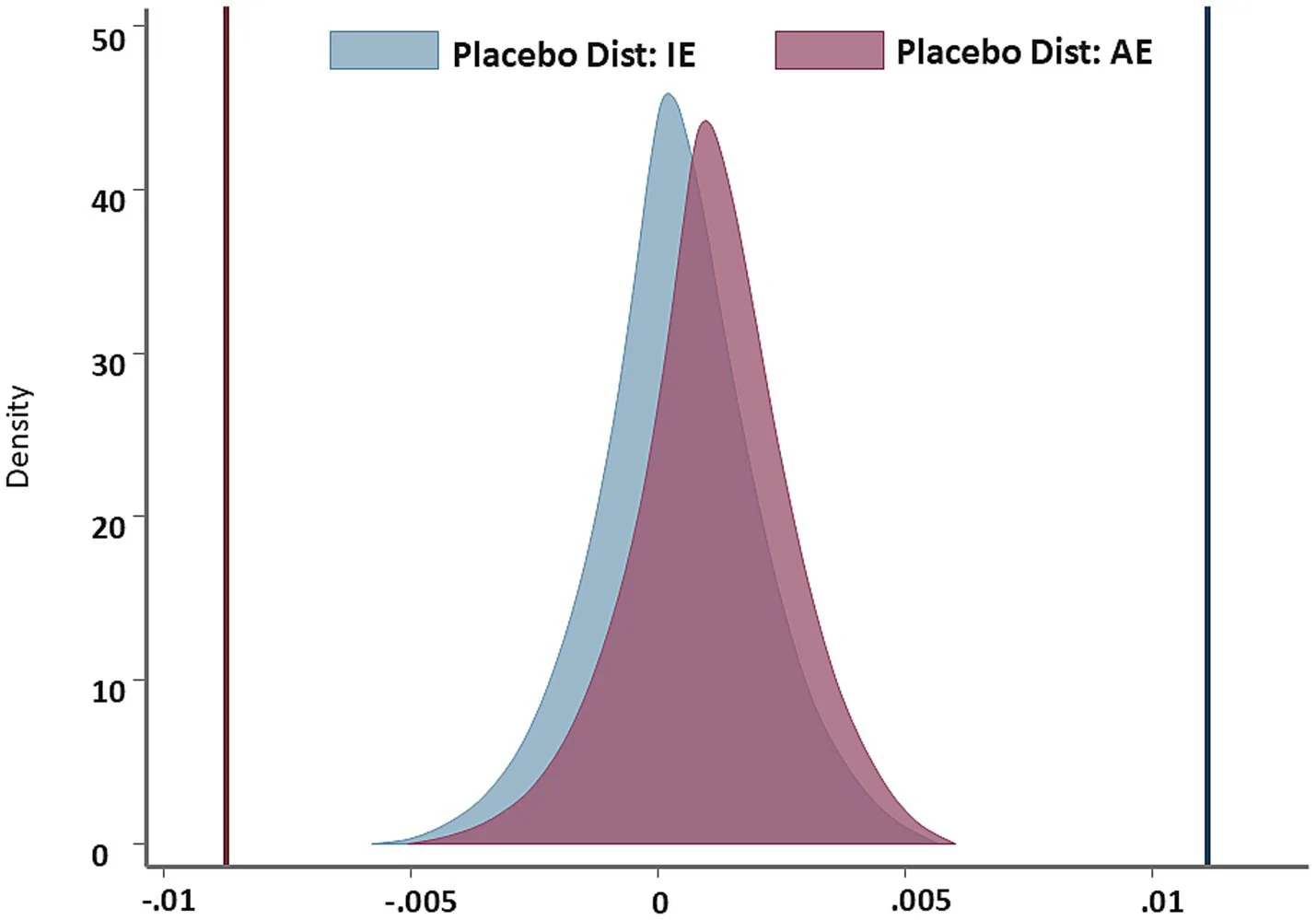
Placebo test curve.

We further conducted placebo tests for robustness analysis. Holding other conditions constant, we randomly permuted IEI and AEI separately and re-estimated the regressions based on the permuted data. For example, keeping AEI and control variables unchanged, we randomly permuted IEI and reran the regression; the same procedure was applied for the placebo test of AEI. Figure 2 displays the kernel density estimation plots from 500 simulations.

The empirical distributions of pseudo-coefficients after random assignment were primarily concentrated around zero. The baseline coefficients for IEI and AEI lay at the far right and far left ends, respectively, showing no obvious relationship with the simulated coefficient distributions. This suggests that the likelihood of the baseline results being driven by random pairing or chance sample fluctuations is low, further enhancing the credibility of the positive association for IEI and negative association for AEI, particularly providing supplementary evidence for H1a.

#### 4.4.5 IV analysis

Drawing on the experience of Guo and Zhu (43), we utilized the community-level mean IEI and AEI levels excluding the individual themselves as instrumental variables (IVs). The digital engagement atmosphere at the community level constitutes an external behavioral norm. This may make individuals more inclined to increase their corresponding types of digital engagement through pathways such as information diffusion and behavioral modeling. In Table 7, the first-stage results, relevant statistics, and reduced-form results all indicate that the IVs meet the relevance requirement. The S-W F statistics indicate that the IVs selected in this study are capable of separately identifying IEI and AEI.

**Table 7:** IV analysis results.

Variables	First stage		Reduced form	2SLS second stage
	IEI	AEI	Depression	Depression
IVIEI	0.142*** (0.022)	0.018 (0.019)	0.026*** (0.008)	
IVAEI	0.008 (0.018)	0.132*** (0.023)	−0.043** (0.021)	
IEI				0.226** (0.094)
AEI				−0.337** (0.167)
S-W F	38.29	29.59		
K-P LM	31.138***			
K-P-W F	13.288			
Baseline controls	Yes	Yes	Yes	Yes
Community-level controls	Yes	Yes	Yes	Yes
TWFE	Yes	Yes	Yes	Yes
*N*	30,751	30,751	30,751	30,751

Standard errors are in parentheses. * *p* < 0.1, ** *p* < 0.05, *** *p* < 0.01. TWFE, Two-Way Fixed Effects.

From the perspective of COR and interactionist stress theories, depression is the result of resource transactions occurring across individual boundaries. High-intensity digital use by other groups in the community constitutes an external stress environment. Unlike macro-environmental factors such as smog, this environment is a symbolic interactive environment. Such environments are usually difficult to associate directly with an individual’s physiological or psychological state, requiring active access by the individual to be converted into an individual load.

It should be noted that the IVs in this study may be subject to reflection problems, meaning that individual behavior and peer behavior might be simultaneously determined or mutually influential. Concurrently, community-level digital engagement may be correlated with factors such as digital infrastructure and urbanization, which could directly affect individual depression. To address this, we excluded the individual when constructing the IVs and incorporated community-level control variables in the 2SLS estimation, such as economic level, average education level, and average employment status. Nevertheless, alternative pathways through which IVs affect individual depression may still exist, and thus this portion of the results should be viewed as a supplementary robustness analysis.

In the second-stage estimation, the association directions of IEI and AEI remained consistent with the baseline results and were both statistically significant at the 5% level. Thus, after employing an alternative identification strategy, the baseline results remained robust.

Overall, although IEI displayed a marginally significant positive association in the benchmark model, it obtained clearer statistical evidence across a series of validation checks. In contrast, the negative association of AEI demonstrated a higher level of consistency. The collective evidence was consistent with H1a and supported H2a.

### 4.5 Exploratory mechanism analysis

Table 8 reports the results of the exploratory mechanism analysis. For IEI, it showed a positive association with psychological mastery resources (0.018), but negative associations with physiological recovery resources such as sleep and exercise (−0.017 and −0.023). Meanwhile, each mediating variable exhibited a statistically significant negative association with depression (b path). The indirect effects for different pathways were approximately −0.006, 0.007, and 0.005, respectively, all statistically significant at the 95% confidence level.

**Table 8:** Exploratory mechanism analysis.

Variables	(1)	(2)	(3)	(4)	(5)
	Digital engagement type	a path	b path	Indirect effect	95% Monte Carlo CI
Future confidence	IEI	0.018** (0.007)	−0.356*** (0.082)	−0.006	[−0.013, −0.001]
	AEI	0.031*** (0.008)		−0.011	[−0.019, −0.004]
Sleep duration	IEI	−0.017** (0.007)	−0.439*** (0.090)	0.007	[0.001, 0.015]
	AEI	−0.009** (0.004)		0.004	[0.001, 0.008]
Exercise duration	IEI	−0.023*** (0.002)	−0.236*** (0.067)	0.005	[0.002, 0.009]
	AEI	−0.016** (0.007)		0.004	[0.0004, 0.008]

Standard errors are in parentheses. * *p* < 0.1, ** *p* < 0.05, *** *p* < 0.01. TWFE, Two-Way Fixed Effects. The indirect effect is the product of the path a and path b coefficients. The 95% Monte Carlo confidence intervals are based on 20,000 random simulations.

AEI positively predicted an increase in psychological mastery resources (0.031), while maintaining negative associations with sleep and exercise (−0.009 and −0.016). Compared to IEI, AEI demonstrated a stronger positive association with psychological mastery resources, but weaker negative associations with physiological recovery resources. The results in Table 8 provide proxy empirical support for the hypotheses of this study.

### 4.6 Boundary condition analyses

#### 4.6.1 Social role stress

As a typical representative of social role stress, the intergenerational caregiving burden faced by the sandwich generation constitutes an external load in individual resource competition. Meanwhile, being part of the sandwich generation is a demographic and life-course objective fact that is not easily reverse-determined by short-term fluctuations in current psychological states. Drawing on the operationalization of the sandwich generation by Lam et al. (44), we categorized individuals aged 30 to 59 years who simultaneously bear childcare and caregiving responsibilities for older adults as having higher role stress, assigning a value of 1, and 0 otherwise. Ultimately, the high role stress group comprised 13,661 observations; descriptive results for the different groups can be found in Appendix Table B.

Table 9 shows that in the high role stress group, the associations of IEI and AEI with depression were stronger, whereas the result for AEI in the low role stress group was not statistically significant. Results for the interaction terms indicate that social role stress amplified the positive association between IEI and depression. However, for AEI, boundary evidence regarding social role stress was not apparent.

**Table 9:** Boundary condition analyses.

Variables	Social role stress	Nonlinear constraints
IEI (Low)	0.009* (0.005)	
AEI (Low)	0.019 (0.026)	
IEI (High)	0.036*** (0.010)	
AEI (High)	−0.045** (0.019)	
IEI × Str	0.027*** (0.008)	
AEI × Str	−0.064 (0.040)	
Wald Test (IEI)	11.391***	
Wald Test (AEI)	2.560	
IEI		0.014** (0.006)
AEI		−0.008** (0.004)
AEI^2^		0.0012** (0.0005)
Controls	Yes	Yes
TWFE	Yes	Yes
*N*	34,198	34,198

Standard errors are in parentheses. * *p* < 0.1, ** *p* < 0.05, *** *p* < 0.01. TWFE, Two-Way Fixed Effects.

#### 4.6.2 Nonlinear constraints of AEI

In Table 9, the coefficients for AEI and its squared term were −0.008 and 0.0012, respectively, both meeting statistical significance tests. This indicates that the negative association between AEI and depression exhibits a U-shaped pattern. Based on calculations from the regression coefficients, the theoretical turning point of this curve corresponds to a sample threshold of 8.933. Combined with the frequency distribution of AEI, approximately 21.8% of the population exceeded this threshold. This result suggests that for the majority of individuals in this study, AEI primarily manifests as psychological restorative engagement. Conversely, a minority of high-intensity users may face risks of resource crowding-out.

## 5 Discussion

### 5.1 The dual and compensatory nature of digital engagement

Synthesizing the results across multiple tests, IE was overall positively associated with depression levels, although its statistical evidence in the baseline model was relatively limited. In contrast, the negative association between AE and depression levels was relatively stable, which aligns with existing findings based on longitudinal data from China (45). It should be noted that the actual effect sizes of both associations were small, suggesting that the linkage between digital engagement and depressive symptoms is a continuous and gradual micro-level connection.

The contrasting associations of IE and AE, to some extent, reflect their differences in real-world contexts. IE is often accompanied by clear deadlines and response requirements. Therefore, high IE not only reflects an individual’s proactive utilization of digital tools, but may also implicitly capture occupational stress and involuntary competitive investment. When digital engagement becomes an extension of real-world tasks, individual autonomy is restricted, and continuous goal management is more likely to encroach upon recovery resources. Consequently, the depression risk associated with IE may not stem entirely from the digital activity itself, as the coercive participation nature and real-world stress behind it also constitute potential explanatory pathways (46, 47). In AE, individuals enjoy greater autonomous space. Recreational involvement encourages individuals to temporarily disengage from real-world demands, offering opportunities for emotional regulation and attentional shift, which aligns with the observed lower depressive tendency (48, 49). This implies that within conventional intensity levels, AE may, through brief psychological detachment, mitigate to some extent the resource depletion brought by real-world competition. Overall, beyond functional orientation differences, the double-edged nature of digital engagement is deeply constrained by the obligational framework, degree of autonomy, and real-world context in which it is embedded.

### 5.2 Explicit benefits and hidden costs

Table 8 provides exploratory evidence concerning the proposed competitive resource pathways. Both IE and AE were associated with increases in psychological mastery resources, while also accompanied by losses in physiological recovery resources. However, the weighting of resource gains and losses exhibits asymmetry between the two. The psychological gains brought by IE were relatively limited, whereas its physiological resource losses were relatively prominent. AE likewise entailed recovery costs, but under conventional participation, its psychological mastery pathway assumed a dominant position.

Regarding IE, efficacy experiences inspired by task advancement may translate into a stronger sense of psychological mastery. However, such dividends are often built upon continuous investment. Existing research has shown that task-oriented digital activities easily permeate into rest periods, compressing basic sleep and physical exercise (50, 51). In this zero-sum game, the loss of physiological recovery resources dilutes part of the psychological gains. Thus, the explicit dividends of IE are often accompanied by hidden recovery costs.

AE presents a different resource structure. Psychological detachment within AE reinforces individuals’ sense of mastery over their own state, which correlates with a lower depression risk. AE similarly occupies the time and space meant for recovery activities, and excessive involvement may also induce fatigue accumulation (52). Nonlinear analyses indicate that AEI for the majority of individuals in the sample fell within empirical threshold limits. This implies that within conventional ranges, the psychological gains of AE hold a relative advantage, yet these gains still face the risk of being offset by resource losses (53).

It should be pointed out that in the psychological mastery resource pathway, confidence in the future may share a certain degree of construct proximity with feelings of helplessness in depressive symptoms. In addition, the core variables relied primarily on self-reports, and individuals’ affective states at the time might have jointly influenced their responses, thereby magnifying statistical associations among variables. On this basis, current results should be viewed as exploratory evidence supporting the competitive resource mechanisms.

Therefore, the difference between IE and AE is not a binary opposition of beneficial versus harmful, but rather a reflection of differences in their underlying resource structures. The resource competition between explicit dividends and hidden costs constitutes an important explanation for the contrasting association directions of IE and AE with depression.

### 5.3 The boundaries of digital engagement

The association between digital engagement and depression is doubly constrained by external role stress and behavioral involvement intensity. Under different conditions, the psychological consequences of the same category of digital activity will diverge.

High social role stress amplified the depression risk associated with IE. Among the sandwich generation, multiple role loads pre-compress individuals’ time and energy (54). In this high-pressure context, external structural demands additionally consume individuals’ limited resources. Coupled with the fact that IE in this group often struggles to foster incremental mastery efficacy, individual resource depletion rises, further tilting the resource balance toward the loss side.

The resource boundaries of AE are constrained by behavioral intensity. Although moderate AE provides emotional compensation for individuals, as involvement intensity increases, this resource balance changes. Nonlinear analyses show that when AEI exceeds the empirical threshold of 8.933, repetitive entertainment leads to diminishing marginal emotional gains. Concurrently, strong digital involvement may cause what was originally a short-term psychological recovery to alienate into persistent avoidance of stress. At this point, the original net resource gain narrows and may even reverse.

Beyond the aforementioned boundaries, resource effects may also be constrained by sociodemographic characteristics, environmental endowments, and digital access conditions. Existing research indicates that stratification by age, gender, and educational attainment systematically reshapes digital access quality, skill barriers, and usage orientations across groups (55, 56). This implies that whether individuals access digital technologies and subsequently lean toward IE or AE is not a random process. Low-frequency digital engagement may simultaneously comprise active restraint and structural exclusion caused by resource scarcity. These two groups exhibit considerable heterogeneity in their socioeconomic positions and disposable resources. This selection mechanism may constrain the average associations observed in this study. Consequently, the findings of this study primarily apply to working-age populations possessing basic digital access capabilities. Identifying the confounding effects of access divides and further delineating resource structure differences across age, occupation, and urban–rural contexts represent important directions for future in-depth research.

### 5.4 Openness of explanatory mechanisms

The competitive resource framework retains theoretical openness. Bidirectional connections may exist between digital engagement and depressive symptoms. Individuals with high baseline depression may be forced to reduce instrumental digital investments due to functional impairment, or may rely more on recreational activities out of an emotional compensation instinct. Concurrently, confounded by common time-varying constraints such as employment changes, health shocks, and financial burdens, the statistical associations observed in this study may reflect a complex interactive structure among digital behaviors, real-world environments, and psychological well-being.

The interpretative boundaries of longitudinal results are also subject to temporal shifts in the underlying meaning of digital behaviors. Although questionnaires across waves maintained similar survey items and coding methods, iterations in platform architectures and algorithmic mechanisms may cause the real-world connotation of isomorphic indicators to continually evolve. Examples include the blurring of functional boundaries in digital behaviors and changes in participation modes and immersion levels within the same category of behavior. This objective drift in behavioral meaning may weaken the strict homogeneity of variables across waves, causing the study results to intertwine genuine individual behavioral adjustments with measurement fluctuations resulting from digital transformations.

Furthermore, potential alternative pathways and exclusion restriction constraints of IVs limit the causal explanatory certainty of their estimates. It should also be noted that high-intensity AE does not equate to genuine problematic use. Problematic use represents a distinct behavior involving loss of behavioral control and daily functional impairment, whose depression-inducing mechanisms often encompass multiple pathways such as self-regulation failure and social function substitution. The competitive resource framework struggles to effectively characterize such complex pathological evolutionary processes. Thus, the empirical threshold of AEI should not be generalized as a clinical diagnostic standard.

Taken together, future research could leverage techniques such as digital footprint tracking and ecological momentary assessment to design more granular variable measurement methods, thereby further disentangling the relationship between resource competition and behavioral choices.

## 6 Conclusion

Grounded in COR theory, this study examined the associations between different categories of digital engagement and depression. The results indicate that IE was overall positively associated with depressive symptoms, whereas AE demonstrated a stable association with lower depression levels, with both associations exhibiting limited effect sizes. Both IE and AE were accompanied by psychological mastery resource gain and physiological recovery resource loss, but the resource loss pathway was more prominent for IE, whereas the resource gain pathway held a relative advantage for AE. In addition, external role stress strengthened the association of IE, while AE offered relatively advantageous psychological benefits within a certain range of behavioral intensity.

Based on these findings, when individuals can hardly avoid digitized work and learning demands entirely, moderate AE can serve as a form of psychological detachment to buffer resource depletion under high-pressure conditions. However, individuals should remain vigilant against the risk of excessive involvement. Particularly for adult populations bearing heavy burdens, there is a need to seek a prudent dynamic balance between psychological gains and physiological costs.

## Data Availability

Publicly available datasets were analyzed in this study. This data can be found: China Family Panel Studies (CFPS), Institute of Social Science Survey (ISSS), Peking University, http://www.isss.pku.edu.cn/cfps/. Data are available to researchers upon registration, institutional verification, and approval through the official CFPS data management platform.
