# Supplementary material for: The divergent impacts of digital engagement on depression: a longitudinal study of competitive resource mechanisms
Source: Front Public Health. 2026 Aug 25;14:1887437. doi: 10.3389/fpubh.2026.1887437 (PMC13549851; doi:10.3389/fpubh.2026.1887437)
Supplement: Supplementary file 1 [file Supplementary_File_1.docx]

**Table A** Construction of Variables

| **Construct** | **Proxy variables** | **Empirical source** | **Coding and construction** | **Role in analysis** |
| --- | --- | --- | --- | --- |
| Depressive symptoms | Depression | CES-D-8 | Eight items summed after reverse-coding two positive-affect items; higher scores indicate higher depressive symptoms; range 8–32 | Outcome |
| Instrumental-oriented digital engagement intensity | IEI | Frequency and importance of Internet use in the domains of work, study, and daily life management | Importance × frequency for each domain; maximum value across Instrumental-oriented domains | Independent variable |
| Affect-oriented digital engagement intensity | AEI | Frequency and importance of Internet use for online games and short-form videos | Importance × frequency for each domain; maximum value across affect-oriented domains | Independent variable |
| Psychological mastery resources | Future confidence | Confidence in the future | Higher values indicate stronger future confidence | Proxy pathway variable |
| Physiological recovery resources | Sleep duration | Usual daily sleep hours | Continuous measure in hours per day | Proxy pathway variable |
|  | Exercise duration | Physical exercise duration | Converted to weekly exercise duration; winsorized at 1st/99th percentiles by year; standardized within year | Proxy pathway variable |
| Sociodemographic covariates | Marital status | Current marital status | 1 = Married/Cohabiting; 0 = Unmarried/Divorced/Widowed. | Covariates |
|  | Employment Status | Current employment status | 1 = Employed; 0 = Unemployed/Out of labor force | Covariates |
|  | Educational Level | Highest level of education attained | Categorical coding based on degree; range 1-8 | Covariates |
| Health behavior covariates | Smoking | Current smoking behavior | 1 = Yes; 0 = No | Covariates |
|  | Alcohol consumption | Current alcohol drinking behavior | 1 = Yes; 0 = No | Covariates |

**Table B** Descriptive Differences Across Subgroups

| **Variables** | **Full Sample (N=34198)** | **Low Role Pressure (Pre=0, N=20537)** | **High Role Pressure (Pre=1, N=13661)** | **SMD** |
| --- | --- | --- | --- | --- |
| Depression | 13.304 | 12.859 | 13.973 | 0.304 |
| IEI | 6.430 | 6.190 | 6.791 | 0.169 |
| AEI | 5.600 | 5.756 | 5.365 | -0.119 |
| Marital Status | 0.791 | 0.738 | 0.871 | 0.328 |
| Employment Status | 0.813 | 0.792 | 0.845 | 0.139 |
| Educational Level | 3.824 | 4.016 | 3.535 | -0.357 |
| Smoking | 0.291 | 0.329 | 0.234 | -0.210 |
| Alcohol Consumption | 0.130 | 0.146 | 0.106 | -0.120 |

**Table 6-1** Sensitivity and Robustness Analyses Results

| **Variables** | **Sensitivity tests** | | **Alternative Classification** | **Cluster Adjustment** | **Period Adjustment** | **LDV model** |
| --- | --- | --- | --- | --- | --- | --- |
|  | **Mean aggregation** | **Sum aggregation** |  |  |  |  |
| IEI | 0.018**  [0.003, 0.035] | 0.022*  [-0.000, 0.045] | 0.022***  [0.011, 0.032] | 0.011*  [-0.001, 0.023] | 0.021**  [0.005, 0.037] | 0.018**  [0.004, 0.032] |
| AEI | -0.012***  [-0.015, -0.007] | -0.015**  [-0.027, -0.004] | -0.028***  [-0.032, -0.024] | -0.009***  [-0.011, -0.006] | -0.027***  [-0.034, -0.018] | -0.011**  [-0.019, -0.003] |
| Depression_t-1_ |  |  |  |  |  | 0.490***  [0.474, 0.506] |
| Controls | YES | YES | YES | YES | YES | YES |
| TWFE | YES | YES | YES | YES | YES | YES |
| N | 34198 | 34198 | 34198 | 34198 | 15038 | 20571 |

Notes: Standard errors are in parentheses. * p<0.1, ** p<0.05, *** p<0.01. TWFE, Two-Way Fixed Effects. 95% CI in brackets.

**Table 7-1** IV Analysis Results

| **Variables** | **First stage** | | **Reduced form** | **2SLS second stage** |
| --- | --- | --- | --- | --- |
|  | **IEI** | **AEI** | **Depression** | **Depression** |
| IV_IEI_ | 0.142***  [0.098, 0.184] | 0.018  [-0.020, 0.054] | 0.026***  [0.011, 0.042] |  |
| IV_AEI_ | 0.008  [-0.027, 0.043] | 0.132***  [0.087, 0.177] | -0.043**  [-0.084, -0.002] |  |
| IEI |  |  |  | 0.226**  [0.041, 0.410] |
| AEI |  |  |  | -0.337**  [-0.664, -0.009] |
| S-W F | 38.29 | 29.59 |  |  |
| K-P LM | 31.138*** | |  |  |
| K-P-W F | 13.288 | |  |  |
| Baseline controls | Yes | Yes | Yes | Yes |
| Community-level controls | Yes | Yes | Yes | Yes |
| TWFE | Yes | Yes | Yes | Yes |
| N | 30751 | 30751 | 30751 | 30751 |

Notes: Standard errors are in parentheses. * p<0.1, ** p<0.05, *** p<0.01. TWFE, Two-Way Fixed Effects. 95% CI in brackets.

**Table 9-1** Boundary Condition Analyses

| **Variables** | **Social role stress** | **Nonlinear Constraints** |
| --- | --- | --- |
| IEI (Low) | 0.009*  [-0.001, 0.019] |  |
| AEI (Low) | 0.019  [-0.032, 0.070] |  |
| IEI (High) | 0.036***  [0.017, 0.056] |  |
| AEI (High) | -0.045**  [-0.083, -0.008] |  |
| IEI×Str | 0.027***  [0.011, 0.043] |  |
| AEI×Str | -0.064  [-0.142, 0.014] |  |
| Wald Test (IEI) | 11.391*** |  |
| Wald Test (AEI) | 2.560 |  |
| IEI |  | 0.014**  [0.002, 0.026] |
| AEI |  | -0.008**  [-0.016, -0.000] |
| AEI^2^ |  | 0.0012**  [0.0002, 0.0022] |
| Controls | YES | YES |
| TWFE | YES | YES |
| N | 34198 | 34198 |

Notes: Standard errors are in parentheses. * p<0.1, ** p<0.05, *** p<0.01. TWFE, Two-Way Fixed Effects. 95% CI in brackets.
